# Endovascular management of giant common iliac artery pseudoaneurysm after complications in simultaneous pancreas–kidney transplant: a case report

**DOI:** 10.1186/s13256-021-02944-w

**Published:** 2021-06-30

**Authors:** Túlio Fabiano de Oliveira Leite, Lucas Vatanabe Pazinato, Thiago Franchi Nunes, Joaquim Mauricio da Motta Leal Filho

**Affiliations:** 1 Interventional Radiologist, Radiology Department, Heart Institute (InCor), University of São Paulo, Rua Rafael Rinaldi, 365 apto 601, São Paulo, Uberlândia 38400-384 Brazil; 2 Interventional Radiologist Unit, Department of Radiology, University of São Paulo Medical School, São Paulo, SP Brazil; 3 Interventional Radiologist, Hospital Universitário Maria Aparecida Pedrossian (HUMAP), Universidade Federal de Mato Grosso do Sul (UFMS), Campo Grande, MS Brazil

**Keywords:** Pancreas–kidney transplantation, Pseudoaneurysm, Endovascular procedures, Visceral transplant pseudoaneurysms, Case report

## Abstract

**Background:**

Pancreatic transplantation is a definitive treatment for selected patients with insulin-dependent diabetes. It is a technically challenging surgery, and vascular complications are the most common cause of pancreatic graft failure. Although rare, pancreas transplants present higher rates of pseudoaneurysms at the vascular anastomosis than other visceral transplants. We present a case of a simultaneous pancreas–kidney transplant complicated with graft failure and common iliac artery pseudoaneurysm that was successfully treated through endovascular techniques.

**Case presentation:**

A 34-year-old White woman presented with abdominal pain and a history of type 1 diabetes mellitus, end-stage renal disease, and two previous pancreas transplantation failures. The first was a simultaneous pancreas–kidney transplantation performed 7 months prior that was complicated by pancreas graft thrombosis within 1 month and required graft resection. Five months later, she underwent a second pancreas transplantation with another pancreatic graft thrombosis requiring graft resection. Abdominal angiotomography revealed a pseudoaneurysm in the right common iliac artery at the point of the previous graft anastomosis. The patient was successfully treated endovascularly with a covered stent in the common iliac artery.

**Conclusion:**

Stent graft implantation for the treatment of common iliac artery pseudoaneurysm as a complication of simultaneous pancreas–kidney transplantation is a safe and feasible procedure.

## Introduction

Simultaneous pancreas–kidney transplantation is a definitive treatment for selected patients with insulin-dependent diabetes and end-stage kidney disease that aims to reduce the long-term complications of both conditions. Pancreatic transplantation is a technically challenging surgery, and vascular complications, such as graft thrombosis, are the most common cause of pancreatic graft failure [1]. Although rare, pancreas transplants present higher rates of pseudoaneurysms at the vascular anastomosis than other visceral transplants, with a reported incidence of 8% [1]. Most reported cases are referred for surgical treatment; however, endovascular approaches have been recently used successfully.

We present a case of simultaneous pancreas–kidney transplant complicated with graft failure and giant common iliac artery pseudoaneurysm that was successfully treated through endovascular techniques. Written informed consent was obtained from the patient for publication of this case report and any accompanying images. A copy of the written consent form is available for review by the Editor-in-Chief of this journal.

## Case presentation

A 34-year-old White woman with type 1 diabetes mellitus diagnosed since youth and end-stage renal disease requiring dialysis therapy over the last 4 years was referred to our hospital with abdominal pain in the right iliac fossa. She had a history of two previous pancreas transplantation failures. The first was a simultaneous pancreas–kidney transplantation performed 7 months prior that was complicated by pancreas graft thrombosis within 1 month and required graft resection. Five months after the first transplant, she underwent a second pancreas transplantation in the right iliac fossa using the same right common iliac artery anastomosis. On the second day postprocedure, her case was complicated by another pancreatic graft thrombosis requiring graft resection. Her renal function was still preserved, but insulin was reintroduced to control glucose levels.

**Fig. 1:**
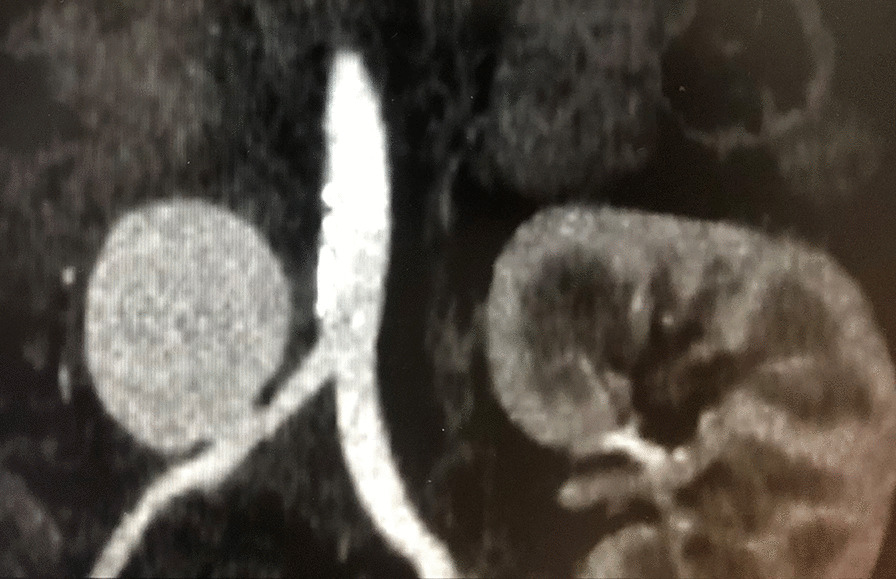
Angiotomography in coronal plane showing a giant right common iliac artery pseudoaneurysm

At our emergency department, she complained of abdominal pain in the right iliac fossa without signs of peritoneal irritation, bleeding, or hemodynamic instability. Abdominal angiotomography was performed and revealed a pseudoaneurysm in the right common iliac artery at the point of the previous graft anastomosis measuring 5.15 × 4.21 × 4.10 cm with a 1.07 × 0.30 cm neck (Fig. 1). There were no signs of active bleeding to the pelvis or hematomas.

**Fig. 2:**
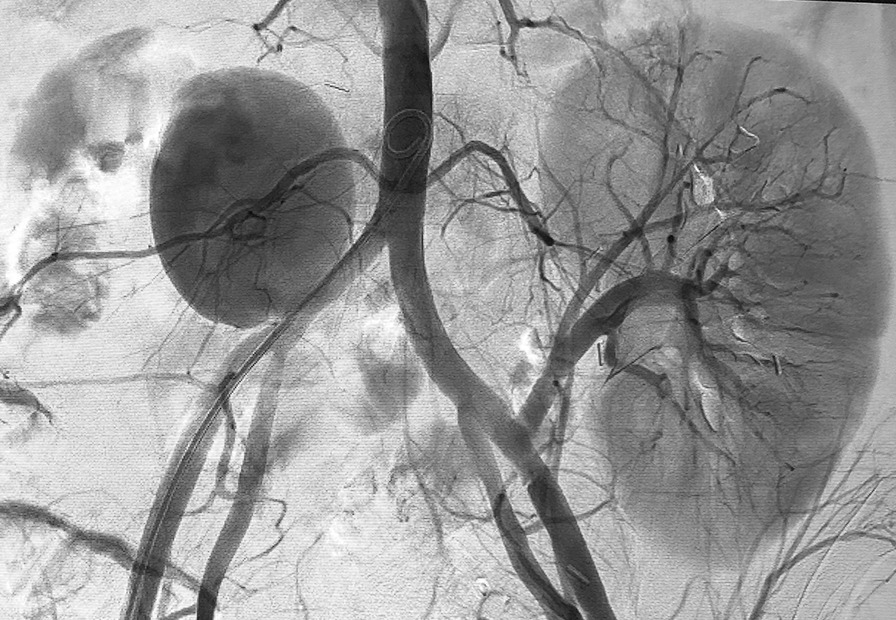
Initial arteriography with visualization of the giant right common iliac artery pseudoaneurysm

**Fig. 3:**
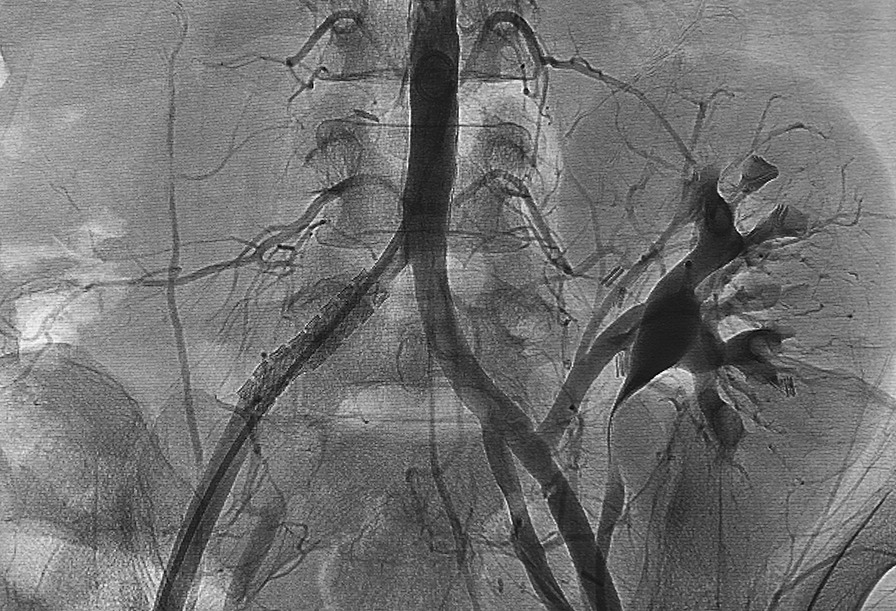
Control arteriography showing the stent graft implanted in the correct location (covering the hole in the right common iliac artery) and pseudoaneurysm exclusion

Endovascular treatment was proposed, and the patient was transferred to our interventional radiology unit. Under local anesthesia and mild sedation, we achieved percutaneous access to the right common femoral artery with a 7-French sheath. On initial arteriography, a right common iliac artery pseudoaneurysm was identified (Fig. 2). To cover the site of extravasation, we implanted a 7 × 39 mm covered balloon-expandable stent (VBX Viabahn W. L. Gore & Associates, Flagstaff, AZ). Control arteriography showed adequate stent location and exclusion of the pseudoaneurysm sac (Fig. 3). An 8-French vascular closure device (Angio-Seal, Terumo, Europe NV) was used for femoral artery sealing, and loading doses of acetylsalicylic acid (ASA) 100 mg and clopidogrel 300 mg followed by daily doses of ASA 100 mg and clopidogrel 75 mg were administered.

The patient was discharged after 2 days with normal renal function and a lack of symptoms. She was followed up for 5 months by interventional radiology and nephrology teams without complaints.

## Discussion and conclusions

Vascular complications are one of the most common and feared complications of visceral transplants, requiring challenging clinical and surgical management [1–4]. Pseudoaneurysms at the anastomosis site are rare but potentially life-threatening conditions and may have multifactorial etiologies such as surgical technique, infection, bile leakage, or pancreatic enzymes [3]. The reported incidence of pseudoaneurysm in pancreatic transplantation, 8%, is higher than those of liver and kidney transplantation (5% and 1%, respectively), and pseudoaneurysms may occur because of laceration or disruption of the arterial wall caused by chemical damage due to exposure to enzymes during pancreatic fistula formation or the development of pancreatic infection, peripancreatic collection, chronic rejection, surgical trauma, or biopsy [1, 5, 6]. Pseudoaneurysms can be related to arterial anastomosis or nonanastomosis, with the former being the most frequent, whereas pseudoaneurysms caused by biopsies usually occur in the pancreatic parenchyma and can lead to arteriovenous fistulas [5]. In our case, the possible causes of pseudoaneurysm formation included multiple previous graft surgeries, remnants of the pancreatic parenchyma releasing proteolytic enzymes, fragility of the vascular anastomoses, friable tissues, and impaired hemostasis.

The clinical presentation of pseudoaneurysms is highly variable, ranging from asymptomatic cases detected on follow-up imaging to the appearance of symptoms such as abdominal pain, endocrine insufficiency, hematuria, palpable and pulsatile masses, hemodynamic instability, and sepsis. They may present as late bleeding, first with mild digestive hemorrhages, called sentinel bleeding, and evolve to an arteriovenous or arterioenteric fistula and eventually to a fatal hemorrhage [5]. Graft control with Doppler ultrasonography at follow-up is important for identifying possible asymptomatic cases [5, 7].

In suspicion of pseudoaneurysm or early complications, ultrasound is usually the diagnostic method of choice, as it is readily available and cost effective [4]. With Doppler ultrasound, pseudoaneurysms are identified as blood-filled lesions; direct communication with the feeding vessel may also be identified, and pulsed-wave Doppler ultrasound may show a “to-and-fro” waveform at the pseudoaneurysm neck. Computed tomography or magnetic resonance imaging is important for diagnosing other complications and planning the proper treatment strategy [5]. Digital subtraction angiography is reserved for cases in which endovascular therapy is chosen.

The decision between endovascular treatment and open surgical repair must be individualized, depending on the location of the lesion, the patient’s clinical status, the presence of abscesses, and the experience of the multidisciplinary team. In some situations, graft resection with deviation of blood flow is necessary to maintain perfusion of the lower limb. In the present case, endovascular treatment was chosen because the patient was hemodynamically stable and had multiple previous open surgeries in the right iliac fossa, which could increase the risk of vascular and intestinal injuries. In addition, whenever feasible, an endovascular approach is preferred as a less invasive alternative with a clinical success rate of 71.4% in patients with hemorrhage after pancreatic transplant and should be considered for first-line therapy in this population [8].

The technique used in the article involved a balloon-expandable stent, although there was no difference between the types of coated expandable or self-expanding stents in the final treatment result. The difference is in the precision in stent release when the balloon is expandable. When there are arteriovenous fistulas, selective microcatheterization and embolization with particles or coils become the options of choice [4]. The use of particles requires experience of the interventional radiologist, as there is a risk of ischemic injury to the pancreas with the use of very small particles. On the other hand, embolization with coils is an excellent option for the treatment of fistulas, especially with controlled release coils and preservation of the collateral circulation. Therefore, the choice of the endovascular technique must take into account the type of injury and the experience of the interventional radiologist.

Pseudoaneurysms, although rare, can be sources of bleeding after pancreas transplantation. The diagnosis requires a high degree of suspicion, and initial imaging is necessary for confirmation and planning of the procedure. Treatment can be endovascular or surgical, depending on the clinical presentation.

## Data Availability

Not applicable.

## Abbreviation

ASA: Acetylsalicylic acid

## References

[1] Lubezky N, Goykhman Y, Nakache R, Kessler A, Baruch R, Katz P, Kori I, Klausner JM, Ben-Haim M. Early and late presentations of graft arterial pseudoaneurysm following pancreatic transplantation. World J Surg. 2013;37(6):1430–7. 10.1007/s00268-013-1972-2 23456259

[2] Arantes RM, Pantanali CAR, Santos VR, D’Albuquerque LAC. Arterial pseudoaneurysm associated with pancreas and kidney transplantation: a case report. Am J Case Rep. 2017;18:198. 10.12659/AJCR.900790 PMC5335644 28232659

[3] Mafeld S, Logue JA, Masson S, Thakkar R, Amer A, Wilson C, Sem G, Manas D, White S, Williams R. Treatment of visceral transplant pseudoaneurysms using physician-modified fenestrated stent grafts: initial experience. Cardiovasc Intervent Radiol. 2019;42(6):920–6. 10.1007/s00270-019-02168-y PMC6502774 30725157

[4] França M, Certo M, Martins L, Varzim P, Teixeira M, Henriques A, Ribeiro A, Alves F. Imaging of pancreas transplantation and its complications. Insights Imaging. 2010;1:329–38. 10.1007/s13244-010-0041-8 PMC3259396 22347926

[5] Ibáñez JM, Robledo AB, López-Andujar R. Late complications of pancreas transplant. World J Transplant. 2020;10(12):404. 10.5500/wjt.v10.i12.404 PMC7769730 33437673

[6] Huurman VAL, Lardenoye JHP. Pancreas graft salvage after successful endovascular treatment of Y graft pseudoaneurysm. J Surg Case Rep. 2019. 10.1093/jscr/rjz124. PMC6521681 31110652

[7] de Oliveira Leite TF, Bortolini E, Linard B, Boueri BA, Carnevale FC, Nomura CH, da Motta Leal Filho JM. Evaluation of morphological and clinical factors related to failure of percutaneous treatment with thrombin injection of femoral pseudoaneurysms from cardiac catheterization. Ann Vasc Surg. 2019;59:173–83. 10.1016/j.avsg.2019.01.013 31028852

[8] Young SJ, Bergren L, Dunn T, Shrestha P, Yadav K, Frank N, Kandaswamy R, Golzarian J. Outcomes of endovascular management of late vascular hemorrhage after pancreatic transplant. Am J Roentgenol. 2018;210(1):201–6. 10.2214/AJR.17.18171 29045177

